# Climate–Water–Food–Nutrition Interaction Across Varying Environmental Contexts: A Population-Representative Analysis of India Data

**DOI:** 10.3390/nu18132045

**Published:** 2026-06-23

**Authors:** Neetu Choudhary, Alexandra Brewis, Amber Wutich, Mihir Kumar Thakur

**Affiliations:** 1 Independent Researcher, Patna 800016, India; 2 School of Human Evolution and Social Change, Arizona State University, Tempe, AZ 85281, USA; alex.brewis@asu.edu (A.B.); amber.wutich@asu.edu (A.W.); 3 Department of Economics, Purnea University, Purnea 854301, India; mihirbhavya@gmail.com

**Keywords:** climate, water, food, nutrition, environment, rainfall, temperature

## Abstract

**Background/Objective**: Achievement of Sustainable Development Goals SDG 2 (child nutrition) depends upon SDG 6 (water insecurity) and SDG 13 (climate action) in multiple ways. However, the current climate–nutrition literature mostly considers water’s effects on nutrition through agriculture and food production. Here, we identify the climate’s impact on child nutrition through its effect on both household food and water security and on their interaction across varying environmental contexts. **Methods**: Using nationally representative data from India, we estimate the climate’s direct association with household water access (time spent fetching water), and both direct and indirect association with household food security (women’s dietary diversity), and child’s dietary diversity and nutrition (HAZ score). Data from 42,567 women and 39,667 children (6–23 months) are analyzed using linear regression and structural equation modeling. **Results**: A unit increase in rainfall is linked to an 18 percent decrease in time to water and an 8.3 percent increase in women’s dietary diversity score. A temperature increase is associated with an increase in time to water and decreased women’s dietary diversity. Time to water mediates the association of temperature and rainfall with women’s dietary diversity, child’s dietary diversity and child’s HAZ score. Households in regions of higher water availability are associated with increased dietary diversity, increased HAZ, and decreased time to water; however, the interaction between climate and regional water availability shows varying effects. **Conclusions**: Climate is associated with household food and water security, which together mediate its association with nutrition. These findings call for broadening the climate action framework to explicitly recognize the multidimensional linkages between SDG 6 and SDG 2.

## 1. Introduction

There is a growing body of theoretical and empirical literature on the climate–nutrition nexus. This literature illustrates how climate change affects overall food security via influences on agricultural productivity and variability in crop yield [1,2,3,4]. These changes in agricultural productivity and food production translate further into lower household socio-economic well-being and food security through both income and price effects [5]. A persistent increase in income volatility due to climate change particularly threatens [6,7] the stability dimension of food security.

Many of climate change’s unfavorable effects are manifested through rises in temperature and rainfall variability. Although rainfall has a mostly positive effect on agriculture [8], rainfall variability affects production adversely [9,10]. Negative rainfall shock (i.e., drought) decreases agricultural productivity, and through it household consumption [5,7,11,12]. Moreover, a rise in temperature hampers agricultural production and micronutrient consumption through its deleterious impact on the nutritional composition and the nutrient availability of certain foods [13,14,15,16]. Even more, water scarcity desolates crop production during times of extreme heat and drought [2,17,18,19]. Extreme weather events in general are found to hamper agricultural and food production. In fact, climate change induced heatwaves and droughts act as catalysts to nutrition insecurity through their adverse impact on both crops and livestock [13,17,20,21,22,23].

Changes in agricultural production, crop yield, or nutrient and micronutrient intake directly affect the efforts towards ’zero hunger’ (SDG 2), one of the 17 Sustainable Development Goals set by the United Nations to be fulfilled by 2030. Importantly, the prevalence of child stunting across sub-Saharan Africa and South Asia is projected to increase due to climate change, particularly rising temperature and excessive rainfall [24,25,26,27]. Generally, an increase in precipitation affects dietary diversity and nutrition positively [28], though it can also lead to poor hygienic practices at home that cause adverse effects [25,29,30,31]. Moreover, a lack of rain may negatively impact child nutrition through, for instance, an elevated risk of vector-borne disease and increased demand on women’s labor [32]. Furthermore, a rise in temperature and its variability can affect dietary diversity adversely [33], though in certain contexts, temperature reportedly has a favorable effect on both agricultural production and nutrition [34,35,36]. Overall, the climatic–nutrition connection is primarily conceptualized and tested through the food availability perspective, with inadequate attention to the role of water.

Climate change’s likely effect on water security (SDG 6) is well recognized [37,38]; however, its subsequent linkage to “zero hunger” (SDG 2) is only partially considered. In fact, climate’s effect on household water security is less examined even while significant impact assessments have focused on household food security [39]. Available studies report the adverse effect of climate change on the overall water situation including its availability, supply, and quality [40,41,42,43], yet the link between the climate and household water insecurity remains largely uninvestigated. To date, changes in both temperature and rainfall levels are known to significantly impact household water security. Glacial recession, caused by climate warming, has led to dry springs [44], increasing time to fetch water or reducing the quantity of water available for household use. A temperature increase of 1 °C is associated with increased daily water collection times of 4 min in a national (sub-regional) analysis [45]. River-dependent households in Botswana reported the negative effect of river flow variability on water security [46]. Even more, households using wells and handpumps face dry period conditions that limit water availability [47]. In contrast, an increase in rainfall has often been associated with better water access. For instance, rainwater harvesting can supply an additional source of water, although with suboptimal drinking quality [47]. Still, excess rainfall can cause mechanical failure in water supply infrastructure and lead to household water insecurity [48]. In a Botswana village, floods have led to the submersion and consequent dysfunctionality of boreholes [46]. Temperature and precipitation together have a significant effect on water collection times, a global indicator of household water insecurity [45].

Household-level water access can also be constrained when regional water availability is high [49]. Irrespective of general water availability, access to a public water supply system is often political. As such, neighborhoods that are not incorporated legally risk being denied due to a lack of rights [46,50]. Increased salinization of ground water due to climate-induced rises in sea levels has become a major source of household water insecurity in certain coastal areas of Bangladesh [51]. High arsenic content in water is another factor contributing to household water insecurity in many areas across the world, irrespective of water availability levels [52].

Water’s effect on nutrition is commonly addressed in terms of its impact on agricultural and food production [6,53]; household-level Water, Sanitation and Hygiene (WASH) mechanisms; and diarrhea among children [54,55]; however, there are other candidate mechanisms through which water can affect child nutrition. For example, being in high-water-availability regions allows for higher availability of water-based animal foods, causing increased dietary diversity [56,57,58]. Water availability also affects consumption preferences, as people may prefer food that uses less water amidst a water-scarce context [59]. Given that water is frequently needed for food preparation [60,61,62], water insecurity affects a child’s dietary diversity and leads to stunting [63,64,65]. Fourthly, women are mostly responsible for food preparation, caregiving as well as water fetching [66,67,68,69], posing competing demands for women’s time and effort and affecting child nutrition adversely. Fifthly, market purchase of water can involve a trade-off against preferred consumption [64,70]. Finally, water is a common need for quenching thirst, better nutrition absorption and other biological functions in the body [71]. Thus, identifying the role of water in the climate–nutrition nexus requires an inquiry into the possible associations between the climate and household water security as well as between household water access and child nutrition through all the above pathways.

Household-level interaction between water and food security remains excluded from the empirical literature. Although these pathways are well recognized, they have yet to be integrated into the broader discourse on nutritionally targeted climate action (SDG 13). This paper tests these pathways empirically by integrating climatic factors, household water and food security and child nutrition across varying contexts in India.

Ranked seventh in the list of countries affected by climate change-driven extreme events in 2019 [72,73], India provides a relevant case study due to wide exposure to climate change and child malnutrition. Furthermore, child health and nutrition in India are ‘most at risk’ to climate change [72]. Additionally, India is experiencing the nation’s most severe water crisis in history as more than 600 million people are exposed to different water stress levels [74]. Evidently climate change, by affecting water availability, has been detrimental to agriculture, traditional livelihoods, access to food, and utilization of food in certain parts of India [75].

The aim of this paper is to elucidate the association of the climate with household water and food security and, in turn, with child nutrition in India while integrating all possible pathways. Using nationally representative data, this paper seeks to explicate how climate variability is associated with child nutrition through both household food and water security.

## 2. Material and Methods

### 2.1. Data and Sample

Our analysis uses aggregated data from India’s Demographic Health Survey (DHS), 2019–2021 [76]. The sample includes 42,567 households/women and 39,667 children aged 6–23 months nested in households. Geospatial covariate data (yearly rainfall in millimeters and yearly temperature in degrees Celsius) has been accessed through DHS’s spatial data repository. This repository contains a standardized set of data obtained from multiple different sources spanning from 2000 to 2020 [77]. This dataset has been linked to standard DHS datasets using common GPS-identified cluster identifiers. Both temperature and rainfall have been standardized with a spatial resolution of 0.5 × 0.5 degrees. Together, these temperature and rainfall data are helpful in capturing climate variability across the country.

### 2.2. The Variables

#### 2.2.1. Outcome Variable

Child’s Height and Age Z (HAZ) score: Calculated as the difference between the individual and the reference population median, divided by the standard deviation of the reference population [78].

#### 2.2.2. Endogenous Outcome Variables

Household water access: Measured by water source, quantity and quality, water-fetching time, or the extent to which household water needs are perceived to be met [79,80]. Furthermore, this paper uses ‘time (spent) to fetch water’ as an indicator of household water access. India’s DHS estimates time to fetch water in minutes per roundtrip, including the time spent waiting. Time to water is a suitable indicator of household water access because it measures both the water access level and women’s time spent fetching water. Water fetching can interfere with other caregiving tasks, since women are the primary caregiver for children [68,69].

Women’s dietary diversity (WDD) score: Proxy for adult household food security. India’s DHS asks women ‘how often do you consume [food]?’ [81]. There are seven food groups considered separately in the questions (i.e., milk or curd, pulse or bean, leafy vegetable, fruit, egg, fish and chicken or meat). In the DHS dataset, the response for each question is coded as 0, 1, 2, 3 for the responses never, daily, weekly, or occasionally, respectively. We recoded the variable, so that a higher number represents a higher frequency of food intake. The maximum possible WDD score is 21. Individuals in a food-secure household, including women and children, have a higher probability of eating diversified food [82,83]. Due to individual heterogeneities and intra-household dynamics, household food security may still be insufficient for guaranteeing individual food security, yet remains necessary. Thus, the association between household food security and women’s dietary diversity is not likely to be linear.

Child’s dietary diversity (CDD) score: Classified as receiving minimum dietary diversity. In the India DHS, respondents are asked whether ‘their child was fed certain foods in the past 24 h’. The response for each question is coded as 0 and 1 for ‘no’ and ‘yes’ respectively. Based on WHO norms [78], these foods are categorized into eight categories: breastmilk; grains, roots and tubers; legumes and nuts; dairy products (milk, yogurt, cheese); flesh foods (meat, fish, poultry and liver/organ meats); eggs; vitamin A-rich fruits and vegetables; and other fruits and vegetables. Thus, a higher score indicates higher CDD level. The maximum possible score is eight after adding all responses.

**Figure 1:**
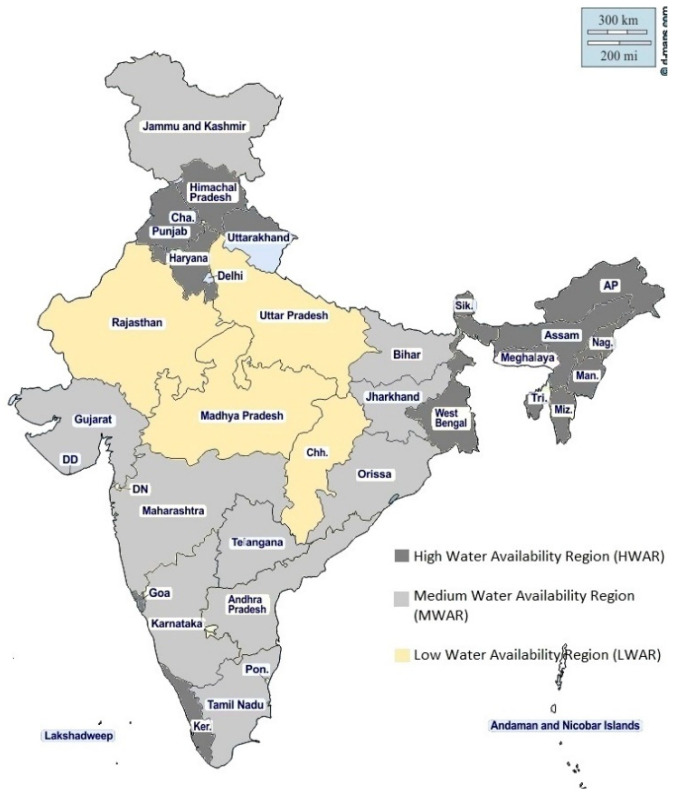
Classification of India based on Water Availability Index. Source: Choudhary et al. 2020 [62].

Key explanatory variables: Two climate variables (i.e., rainfall and maximum temperature) were obtained from the DHS spatial data repository and employed as key covariates [77]. Our model further implements a spatial classification system for India to account for regional environmental differences. Water availability is a critical environmental indicator used to reflect ecosystem health, such as biodiversity and forestation [84]. The Surface Water Availability Index [85] classifies India into five regional groups with different water availabilities (excluding locations without data), recategorized into three groups for our analysis (see Figure 1): low-water-availability regions (LWARs), medium-water-availability regions (MWARs), and high-water-availability regions (HWARs). HWARs include all north-eastern Indian states (with the exception of Arunachal Pradesh), along with West Bengal, Himachal Pradesh, Punjab, Haryana, Uttarakhand, Kerala, and Goa. MWARs include the states of Bihar, Jharkhand, Odisha, Andhra Pradesh, Tamil Nadu, Maharashtra, Karnataka, Jammu and Kashmir, and Gujarat. LWARs include Telangana, Arunachal Pradesh, Madhya Pradesh, Chhattisgarh and Uttar Pradesh.

Our model accounts for seasonality by creating a unique variable to reflect the season that the survey was taken in. This variable has four seasonal categories as determined by the Indian Meteorological Department [86]: pre-monsoon (March–May), monsoon (June–September), post-monsoon (October–December) and winter monsoon (January–February) season. 

Other explanatory variables: The DHS’s standard household wealth index includes water access. To reduce the potential of confounding, we developed an alternative asset score to measure household material wealth that excluded water, based on the method described by Rutstein and Johnson [87]. In our model, this alternative household asset score is a continuous variable. Household access to improved toilet facilities [88] is an indicator of household sanitation. Additional explanatory variables include maternal education, household demography, and child-specific characteristics related to health and demography.

### 2.3. Conceptual Framework and Estimation Strategy

**Figure 2:**
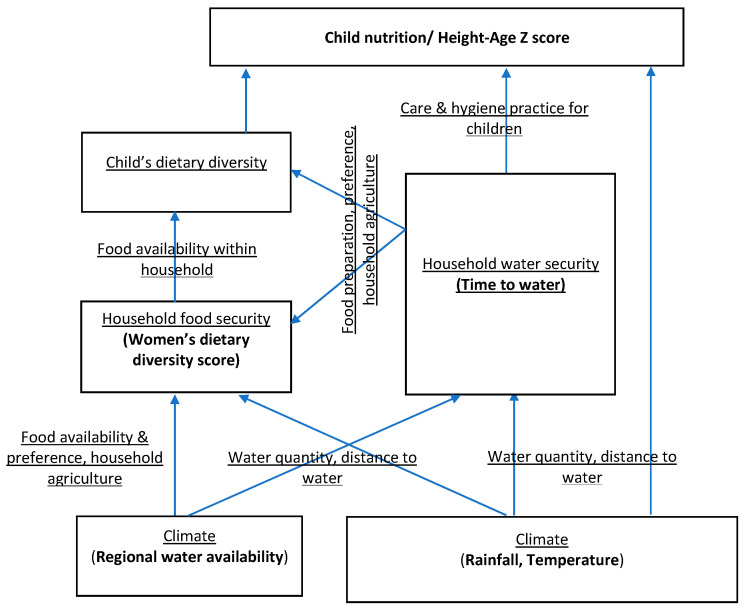
Conceptual framework on water, food and nutrition interaction in climate change context. Source: Authors’ construct.

The conceptual framework depicted in Figure 2 describes how climate and regional water availability impact household water security. Down the line, child nutrition is affected by household food security, child’s dietary diversity and care practices. Thus, the effect of rainfall and temperature on child nutrition is mediated by household water security through multiple pathways. Furthermore, climatic factors, particularly rainfall variability, interact with regional water availability to produce varying child nutrition outcomes.

Multiple linear regression was used to identify the association between climatic factors and household water and food security (Model 1);

Y_tot_ = α + β (X) + γ(X_1_) ε_1_, (1)

Y_hfs_ = α + β (X) + γ(X_1_) ε_1_, (2)

where Y_tot_ = time to water in minutes, Y_hfs_ = WDD score, X_1_ = climatic variables, X = other covariates.

To assess the mediating role of the three endogenous variables (i.e., household water access, WDD and CDD), we use structural equation modeling (SEM). This approach can be practical when there are endogenous explanatory variables, including linked regression style equations [89], particularly when multiple mediators are present in the model [90,91]. The SEM analysis based on the following linear equations is the foundation of our analysis (Model 2):

Y_1_ = α_1_ + β_1_ Y_2_ + μ_1_Y_4_ + λ_1_ (X_1_) +δ_1_ (X) + ε_1_ (3)

Y_2_ = α_2_ + β_2_ (Y_3_) + μ_2_ (Y_4_) + δ_2_ (X) + ε_2_ (4)

Y_3_ = α_3_ + β_3_ (Y_4_) +μ_3_ (X_1_) + λ_3_ (X_2_) + δ_3_ (X) + ε_3_. (5)

Y_4_ = α_4_ + β_4_ (X_1_) + μ_4_ (X_2_) + δ_4_ (X) + ε_4_ (6)

Here Y_1_ represents child’s HAZ score; Y_2_ represents (CDD) score; Y_3_ represents WDD score; Y_4_ represents time to water in minutes; X_1_ represents climatic variables; X_2_ represents regional water availability; and X represents other covariates.

Our final model (3) includes the interaction effect between climatic variables and regional water availability. For this we replace Equations (5) and (6) from Model 2 with Equations (7) and (8) below:

Y_3_ = α_3_ + β_3_ (Y_4_) +μ_3_ (X_1_) + λ_3_ (X_2_) + δ_2_ (X) + δ_3_ (X_2_) (X_1_) + ε_3_ (7)

Y_4_ = α_4_ + β_4_ (X_1_) + μ_4_ (X_2_) + λ_4_ (X) + δ_4_ (X_2_) (X_1_) + ε_4_ (8)

All analyses accounted for survey characteristics, such as clustering, stratification and sample weight using the svy command in STATA 16. Our model is identified as having a unique solution for all the parameters. Also, the sem command in STATA automatically checks for model identification.

## 3. Results

**Figure 3:**
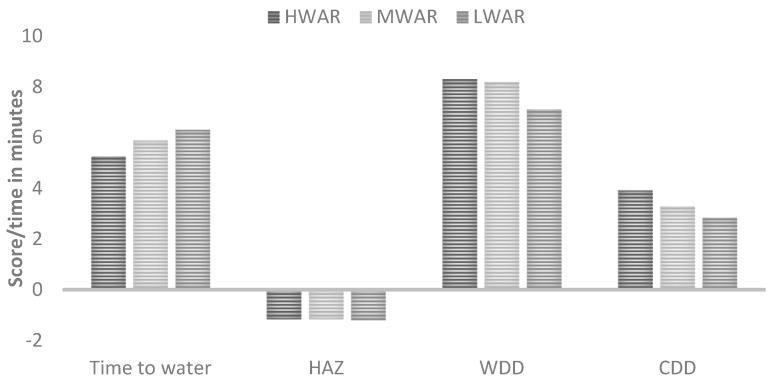
Mean time to water, mean HAZ scores, mean CDD scores, and mean WDD scores across water availability regions. Source: Authors’ construct.

Sample characteristics are given in the Supplementary Table S1. Figure 3 exhibits mean HAZ, WDD, and CDD scores, in addition to mean time to water by regional water availability levels. Mean time to water is lowest in HWARs and highest in LWARs, whereas mean HAZ, CDD, and WDD scores are highest in HWARs and lowest in LWARs.

### 3.1. Climate Affects Both Household Food and Water Security

Table 1 presents results from Model 1 using linear regression. A logarithmic unit increase in rain is associated with an 18% decrease in time to water and a unit increase in maximum temperature is associated with approximately a 17% increase in WDD score, at 0.01 level of significance (α = 0.01). A logarithmic increase in rain is further associated with an 8.3% increase in household food security. Similarly, an increase in temperature is linked with a 4.7% decrease in WDD score (α = 0.01). Altogether, these shifts show that time to water is proportionately lower during the monsoon, post-monsoon and winter monsoon season than during the pre-monsoon season. Moreover, WDD is higher during the monsoon and post-monsoon seasons compared to the pre-monsoon season by approximately 38% and 64%, respectively at α = 0.01. During the winter monsoon season, WDD is higher by 9.5% at α = 0.05.

Increasing a woman’s years of education leads to a 9.4% decline in time to water and a 1.6% increase in WDD score at α = 0.01. Increases in asset score are also associated with lowered time to water and an increased WDD at α = 0.01. Rural households are associated with an increased time to water and decreased WDD. Identifying as Muslim is associated with lower time to water and higher WDD scores at α = 0.01, compared to Hindu households. Caste also appears significant at α = 0.01. Among castes, being in an ST household is associated with increased time to water and WDD scores as compared to an SC household; meanwhile, OBCs or general caste households are associated with reduced time to water and increased WDD scores. MWAR and LWAR households are associated with higher time to water, relative to HWARs at α = 0.01; however, LWAR households are associated with lower WDD scores.

### 3.2. Household Water Insecurity Mediates Climate’s Association with Household Food Security and Climate’s Association with Child Nutrition

SEM estimates from the mediation analysis (Model 2) are presented in Table 2. This model explores the mediating roles of WDD, CDD, and time to water in climate–nutrition interactions. Similar to our linear regression model (Table 1), temperature and rainfall are associated with both time to water and WDD at α = 0.01. An increase in temperature and rainfall is associated with an increase and decrease in time to water, respectively. Temperature and rainfall are also associated with CDD score at α = 0.01. MWAR and LWAR households are associated with increased time to water by 34% and 56% at α = 0.01, respectively. An increase of one minute in time to water is associated with a 2% decrease in WDD scores. In addition, increased rain and increased temperature are directly associated with an increase and decrease respectively in WDD score at α = 0.01. MWARss are associated with increased WDD scores; meanwhile, LWARs are associated with decreased WDD scores by 55% α = 0.01 and 32% at α = 0.050.

An increase in time to water is linked with a 1.3% decrease in CDD scores at α = 0.01 (Table 2). A unit increase in WDD scores is linked with an increase of 13.4% in CDD scores at α = 0.01. An increase in time to water of one minute is associated with an approximate 1% decrease in child HAZ scores and an increase in CDD scores is associated with increased HAZ score by 7%, both at α = 0.01. A logarithmic increase in rainfall is associated with a 1% increase in HAZ score at α = 0.05 and an increase in temperature causes around a 2% decline in HAZ score at α = 0.01. Compared to the pre-monsoon season, child HAZ scores are 6% and 13% lower in the monsoon and post-monsoon period respectively at α = 0.01. In contrast, CDD scores are increased by approximately 21% and 11% at α = 0.01 during the corresponding periods. Child HAZ scores are positively associated with access to toilet and safe disposal of child’s stool, both at α = 0.01. In addition, various socio-economic and demographic variables are also associated significantly with child HAZ score.

Table 3 shows the direct and indirect effects of rainfall and temperature on WDD, CDD, and child HAZ scores. Rainfall and temperature have an indirect association with these three outcomes at α = 0.01. Rainfall does not have a significant direct association with HAZ score. Moreover, 79% of its effect on HAZ score is mediated through time to water and CDD score. The percentage contributions of rainfall’s indirect effect on WDD and CDD scores are 44% and 26%, respectively. The indirect percentage contributions of maximum temperature are smaller, yet significant, at 33%, 22% and 1.9% for HAZ score, CDD score and WDD score respectively.

### 3.3. Climate Interacts with Environmental Context to Produce Varying Nutrition Outcomes

SEM results from our mediation analysis incorporating the interaction effect between regional water availability and climate (i.e., rainfall and temperature) are given in Table 4. Compared to the logarithmic unit increase in rain across HWARs, an increase in rainfall in MWARs is not significantly associated with time to water; however, a unit increase in rainfall in LWARs is linked with a 62% rise in time to water at α = 0.01. A temperature increase in LWARs is further linked with a 21% increase in time to water at α = 0.01. Additionally, a logarithmic unit increase in rain across MWARs is associated with a 19% increase in WDD score at α = 0.01; however, an increase in rainfall across LWARs is not associated with a significant change in WDD scores. An increase in temperature in both MWARs and LWARs is associated with a 4.5% and 2% decline in WDD score at α = 0.01. An increase in WDD score is significantly associated with a 13.5% increase in CDD score at α = 0.01.

The interaction effect of the water availability region and rainfall on a child’s HAZ is also significant at α = 0.01. An increase in rainfall in MWARs and LWARs is associated respectively with a 9.3% and a 4.8% increase in child HAZ scores. Furthermore, an increase in temperature in MWARs and LWARs causes a 2.7% and 1.4% decline in HAZ scores at α = 0.01. Notably, an increase in time to water remains associated with a reduction in HAZ score by 1.2%, though we have controlled for CDD score and climatic variables; and a unit increase in CDD score is linked to a 1.7% increase in child’s HAZ score, both at α = 0.01.

**Figure 4:**
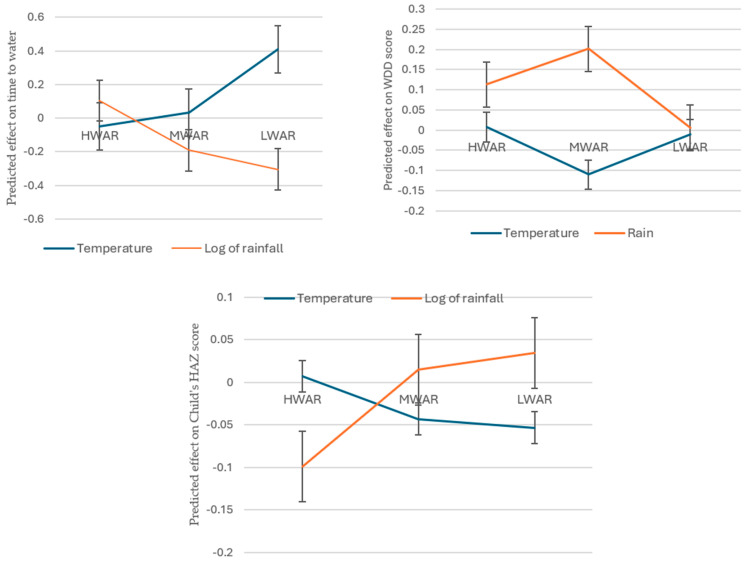
Predicted effect of temperature and rainfall on time to water, WDD score and HAZ score (α = 0.01). Source: Authors’ construct.

In Figure 4, the predicted effects associated with logarithmic unit increases in rainfall and maximum temperature on time to water and child’s HAZ score are presented, at α = 0.01. In HWARs, a temperature increase predicts an increase in HAZ scores; however, it suggests a decline in HAZ scores across MWARs and LWARs. The predicted impact associated with an increase in rainfall is also negative in HWARs and positive across MWARs and LWARs. A unit increase in temperature is associated with a predicted decrease in time to water in HWARs, and increase in MWARs and LWARs. In addition, increased rainfall is associated with an increase in time to water across HWARs and a decrease in MWARs and LWARs. Moreover, an increase in rainfall is predicted to be associated with increased WDD scores in HWARs and decreased WDD scores in LWARs. An increase in temperature is further predicted to be linked to an decrease in WDD scores across MWARs and LWARs. Temperature and rainfall in LWARs have the highest size effect.

## 4. Discussion

Using nationally representative data from India’s DHS program, this analysis suggests pathways of impact from the climate (rainfall and temperature) on household water access (time to water), household food security (WDD) and child nutrition (HAZ). We further identify the mediating roles of household water insecurity that interface with food security (WDD score). The mediating roles of both household water access (time to water) and household food security (WDD) in underscoring the climate’s impact on child dietary diversity (CDD) and growth (HAZ score) are also identified. Our findings explicate the less-explored terrain of water and food interactions, as well as their mutually reinforcing role in defining the climate’s impact on nutrition.

Our analysis shows that rainfall and temperature are significantly associated with household food security and water access, as reflected by changes in WDD and time spent accessing water respectively. This aligns with the available literature linking the climate to water and food security [1,2,8,11,45,46,47].

The mediation analysis describes the direct and indirect channels through which the climate interrupts household food and nutrition security. An increase in rainfall and temperature manifests in direct positive and negative associations with child nutrition, respectively. In addition, there are indirect associations through their links with household water and food security. This is a novel finding, given that previous studies have only examined the water–nutrition connection through food security [92,93,94,95]. Moreover, household water security, influenced by climate change, connects with child nutrition through several mechanisms other than sanitation and hygiene as emphasized in the recent literature [96,97,98,99]. Our findings also show that household water access is linked to child nutrition through its association with household food security. This aligns with recent findings linking household water access with dietary diversity and nutrition among children [62,63]. Mothers must manage competing demands for time; thus, water access is connected directly with a child’s dietary diversity [62]. Our findings further confirm previous evidence corroborating the negative effect of heat stress and drought on dietary intake and nutrition [100,101,102,103,104,105,106]. Furthermore, seasonality has an association with child nutrition. SEM findings show that monsoon season (June–September) is associated with decreased HAZ scores, although CDD increases during periods of monsoon. Increased precipitation, particularly during the monsoon season, was previously found to be associated with an increase in child undernutrition, possibly due to increased diarrhea exposure and a lack of hygiene [25,107]. Previously, seasonality associated with monsoons has been found to affect both the child’s dietary diversity and anthropometric outcomes [25,107,108].

Our models also included regional environmental contexts (water availability), which were found to be associated with child nutrition through both household food and water security. Being in medium-water-availability regions (MWARs) and low-water-availability regions (LWARs) is associated with lower household food, water and nutritional security, as compared to high-water-availability regions (HWARs). This is possible because greater water availability in HWARs creates opportunities to access diversified foods, such as through household agriculture, fishing and other related water-dependent activities [56,58,59]. Households in HWARs may also be conducive to enhanced water access while a lack of water can constrain the same [109,110]. This in turn has implications for child nutrition.

Results from Model (3) show that climatic variables interact with regional water availability to connect with varying food, water and nutritional security outcomes. When compared to HWARs, an increase in rainfall across LWARs is significantly associated with reduced household water insecurity. It is possible that in water-rich areas, increases in rainfall cause excess water in the environment, potentially causing waterlogging that disrupts water supply infrastructure among others [48]. Excess rain has been found to cause mechanical failure in water supply systems [46]. On the other hand, an increase in rainfall in LWARs may cause improved water access [47]. The predicted effect-size of rainfall is also negative for child nutrition in HWARs, possibly due to an increased incidence of diarrheal disease caused by excess water [29,31]. The predicted effect-size of rainfall is positive on HAZ scores in LWARs. This is possibly because a rise in rainfall, and consequent improved water access in LWARs allows for preparation and consumption of a more diversified diet, thus leading to improved nutrition [27]. This aligns with the existing literature [27,28,106], linking increases in rainfall with improved CDD and HAZ. An increase in rainfall in LWARs does not have a significant association with household food security; however, it is associated with increased food security levels in MWARs. An increase in temperature in both MWARs and LWARs is associated with reduced food security. This is ostensibly because additional heat is further likely to dry up water supply sources in water-scarce contexts.

Our findings have policy implications for food, water and nutrition security under climate change conditions. Across India, we observe that rises in temperature and frequent variability in rainfall are associated with reduced household food, water and nutrition in LWARs. Therefore, these regions might particularly benefit from nutrition-sensitive social protection programs. India has a number of these support programs, such as the Integrated Child Development Services (ICDS). Nevertheless, these programs remain uniform across the nation and do not receive context-specific support against climatic factors or water insecurity. For instance, a lack of water was identified as a major challenge facing ICDS, as water sources are often far from ICDS centers [111]. Such a challenge is exacerbated by rising temperatures, particularly in LWARs. Moreover, HWARs need better water management policies, as heavy rainfall can be detrimental to water and nutrition security efforts in these regions. In water-rich areas, flood management requires oversight of water run-off. As such, check dams, ditches, and other infrastructural components are constructed to minimize the impact of excess rainfall. The leading flood management practices in India lack context and community participation, and, instead, focus on structural measures to fight and control floods [112].

Our analysis underscores the importance of a broader SDG 13 framework for effective climate action. Such a framework would further unravel the underlying mechanisms of the climate’s (i.e., rainfall and temperature) effect on household food and water security (SDG 6) and child nutrition (SDG 2). Climate mitigation and adaptation policies adopted under SDG 13 must be responsive to the climate’s effect on household food and water security. Even more, SDG 13 should be made nutrition-sensitive while considering the multiple pathways that connect water to nutrition. Furthermore, context-specific policies will be essential for navigating the climate’s regional variation and impact on nutrition. Current policies solely consider the climate’s impact on nutrition through agriculture, neglecting alternative pathways linking the climate and nutrition. We find that water–nutrition interactions are multidimensional, wherein child dietary diversity and WASH mediate the association between household access to water and child nutrition. Identifying dietary diversity as a pathway provides nutrition policy with an additional window of intervention, particularly in LWARs. Ultimately, our analysis measures household water security by time to water, which offers a gender-sensitive opportunity to study maternal roles in the climate–nutrition interactions that affect children. Climate and nutrition policies in India have yet to reflect mutual interconnectedness.

### Limitations

Our analysis is based on cross-sectional data and, therefore, cannot demonstrate causality. While assessing household water security, we could not account for water quality. The DHS data collection took place in 2019–2021, and hence spanned COVID-19. Given the well-documented interactions between COVID-19 and water insecurity [49], we acknowledge this may be reflected in our results. The association of climate–food–water–nutrition may possibly become more pronounced, if the effect of COVID-19 is accounted for in the model. This is because the pandemic was accompanied by prolonged lockdowns, which disrupted access to water sources external to households while consumption demand grew due to sanitary and hygiene needs [113].

## 5. Conclusions

Using nationally representative data from India, this work describes the household-level interaction between Sustainable Developmental Goals 2 and 6 under climate change contexts across regions with varying environmental contexts. Our findings ultimately support broadening SDG 13 frameworks to further integrate household water security.

## Figures and Tables

**Table 1:** Estimates from linear regression on effect of climate on household water and food security [N = 42,567].

MODEL 1	Household Water Security (Time to Water)		Household Food Security (WDD Score)	
Model 1 [N = 42,567]				
	Coef.	Std. Err.	Coef.	Std. Err.
Log rain	−0.1822 **	0.0681	0.0836 **	0.0130
Maximum temperature	0.1763 **	0.0276	−0.0466 **	0.0042
Water availability region (ref: HWAR)				
MWAR	0.3660 **	0.1700	0.5532 **	0.0251
LWAR	0.6017 **	0.1709	−0.3225 **	0.0252
Season (ref: pre-monsoon)				
Monsoon	−1.27 **	0.2098	0.3821 **	0.0388
Post-monsoon	−1.59 **	0.1892	0.6393 **	0.0395
Winter monsoon	−0.4052 *	0.1912	0.0955 *	0.0351
Asset score	−1.00 **	0.0670	0.1424 **	0.0141
Female’s highest year of education	−0.0941 **	0.0319	0.0168 **	0.0073
Religion (ref: Hindu)				
Muslim	−0.5001 **	0.1544	0.9307 **	0.0400
Christian	0.6731	0.4471	0.4922 **	0.0652
Other	−0.2420	0.4237	−0.4885 **	0.0736
Caste (ref: SC)				
ST	2.27 **	0.3369	−0.2512 **	0.0417
OBC	−0.5627 **	0.1478	0.3059 **	0.0318
General	−0.5535 **	0.1879	0.6529 **	0.0401
Residence (ref: urban)				
Rural	1.068 **	0.1328	−0.2432 **	0.0322
Gender of head of household (ref: male)				
Female	−0.2183	0.14425	0.0828 **	0.0323
Model fit				
F(22, 17,565) = 52.06 ** R-squared = 0.0641				

** significant at 0.01 level; * significant at 0.05 level.

**Table 2:** SEM estimates on impact of climate change on child’s HAZ (Model 2).

HAZ Score		CDD Score		WDD Score		Time to Water	
MODEL 2 (N= 39,667)							
	Coef. & Std. Err.		Coef. & Std. Err.		Coef. & Std. Err.		Coef. & Std. Err.
Time to water	−0.0091 ** 0.0008	Log of rain	0.0696 ** 0.0093	Log of rain	0.0549 ** 0.0086	Log of rain	−0.2180 ** 0.0589
Log of rain	0.0106 * 0.0096	Max temperature	−0.0370 ** 0.0025	Max temperature	−0.0621 ** 0.0025	Max temperature	0.0777 0.0171
Max temperature	−0.0249 ** 0.0026	Time to water	−0.0133 ** 0.0008	Season (ref: pre-monsoon)		Season (ref: pre-monsoon)	
Season (ref: pre-monsoon)		WDD score	0.1349 ** 0.0052	Monsoon	0.3894 ** 0.0283	Monsoon	−1.73 ** 0.1936
Monsoon	−0.0626 ** 0.0296	Asset score	0.0636 ** 0.0108	Post-monsoon	0.5984 ** 0.0281	Post-monsoon	−1.80 ** 0.1920
Post-monsoon	−0.131 ** 0.0302	Season (ref: pre-monsoon)		Winter	0.0628 ** 0.0252	Winter	−0.6235 ** 0.1723
Winter	−0.0031 0.0284	Monsoon	0.2097 ** 0.0286	Water availability region (ref: HWAR)		Regional water availability (ref: HWAR)	
CDD score	0.0710 ** 0.0051	Post-monsoon	0.1126 ** 0.0295	MWAR	0.5540 ** 0.0253	MWAR	0.3480 * 0.1716
Gender of head of household		Winter	0.0414 0.0274	LWAR	−0.3171 ** 0.0254	LWAR	0.5670 ** 0.17262
Female	−0.0953 ** 0.0263	No. of children under five	−0.0624 ** 0.0110	Time to water	−0.0207 ** 0.0007		
Gender of child		Gender of head of household					
Female	0.2427 ** 0.0187	Female	0.0674 ** 0.0255				
Age of child	−0.0721 ** 0.0018	Gender of child					
Access to WHO toilet (ref: no)		Female	−0.0197 0.0181				
Yes	0.0837 ** 0.0222	Age of child	0.0871 ** 0.0017				
Safe disposal of child’s stool (ref: no)		Residence (ref: urban)					
Yes	0.0808 ** 0.0183	Rural	0.0398 ** 0.0246				
Caste (ref: SC)		Female’s highest year of education	0.0035 ** 0.0054				
Schedule tribe	0.1039 ** 0.0288	Religion (ref: Hindu)					
OBC	0.1157 ** 0.0229	Muslim	0.0496 0.0321				
General	0.2965 ** 0.0287	Christian	0.5939 ** 0.0377				
Had diarrhea [ref: no]		Other	0.1581 ** 0.0454				
Yes	−0.0678 ** 0.0278	Caste (ref: SC)	.				
Residence (ref: urban)		Schedule tribe	0.1748 ** 0.0323				
Rural	−0.0112 0.0235	OBC	−0.0368 0.0249				
Asset score		General	0.0617 ** 0.0303				
Maternal education	0.0172 ** 0.0056						
Religion (ref: Hindu)							
Muslim	−0.0192 0.0280						
Christian	0.1928 ** 0.0359						
Other	0.1245 ** 0.0435						
Model fit							
Likelihood ratio (model vs. saturated): chi2_(19) = 467.97 ** Comparative fit index = 0.967							

** significant at 0.01 level; * significant at 0.05 level.

**Table 3:** Direct and indirect effect of climate on WDD score, CDD score and child’s HAZ score.

HAZ Score	Direct Effect (Coef. & Std. Err.)	Indirect Effect (Through Time to Water and CDD Score (Coef. & Std. Err.)	Total Effect (Coef. & Std. Err.)	Percentage Contribution of Mediated Effect
Log of rain	0.0022 0.0102	0.0084 ** 0.0096	0.0106 * 0.0096	79
Max temperature	−0.0161 ** 0.0026	−0.0083 ** 0.0008	−0.0249 ** 0.0026	33
CDD score	Direct effect	Indirect effect (through time to water and WDD score)	Total effect	
Log of rain	0.0513 ** 0.0093	0.0183 ** 0.0011	0.0696 ** 0.0093	26
Max temperature	−0.0292 ** 0.0025	−0.0078 ** 0.0004	−0.0370 ** 0.0025	21
WDD score	Direct effect	Indirect effect (through time to water)	Total effect	
Log of rain	0.0305 ** 0.0086	0.0244 ** 0.0003	0.0549 ** 0.0086	44
Max temperature	−0.0612 ** 0.00251	−0.0012 ** 0.0001	−0.0624 ** 0.0025	1.9

** significant at 0.01 level; * significant at 0.05 level.

**Table 4:** SEM results for impact of climate change (Table 4) on HAZ including the interaction effect (Model 3).

HAZ Score		CDD Score		WDD Score		Time to Water	
Model 3 (N= 39,667)							
	Coef. & Std. Err.		Coef. & Std. Err.		Coef. & Std. Err.		Coef. & Std. Err.
Time to water	−0.0121 ** 0.000829	Time to water	0.0103 * 0.0008	Time to water	−0.3913 ** 0.0591	Water availability regions and rain [ref: HWAR# Log of rain]	
CDD score	0.0171 ** 0.0051	WDD score	0.1349 ** 0.0052	Water availability regions [ref: HWAR# Log of rain]		MWAR # Log of rain	0.0388 0.0601
Water availability regions and rain [ref: HWAR# Log of rain]				MWAR# Log of rain	0.1949 ** 0.0088	LWAR # Log of rain	−0.6203 ** 0.0709
MWAR# Log of rain	0.0935 ** 0.0099			LWAR # Log of rain	0.0060 0.0104	Water availability regions and temperature [ref: HWAR# temperature]	
LWAR # Log of rain	0.0489 ** 0.0117			Water availability regions [ref: HWAR# temperature]		MWAR# temperature	0.0157 0.0169
Water availability regions and temperature [ref: HWAR# temperature]				MWAR# temperature	−0.0452 ** 0.0025	LWAR# temperature	0.2097 ** 0.0205
MWAR# temperature	−0.0269 ** 0.0028			LWAR# temperature	−0.0208 ** 0.0030		
LWAR# temperature	−0.0142 ** 0.0034						
Model fit							
Likelihood ratio (model vs. saturated): chi2_(20) = 636.32 ** Comparative fit index = 0.957							

** significant at 0.01 level; * significant at 0.05 level.

## Data Availability

The article uses Demographic and Health Survey data. This data is freely downloadable from www.dhsprogram.com, after placing a request.

## Supplementary Materials

The following supporting information can be downloaded at: https://www.mdpi.com/article/10.3390/nu18132045/s1, Table S1: Sample characteristics.
