# Supplementary material for: Climate–Water–Food–Nutrition Interaction Across Varying Environmental Contexts: A Population-Representative Analysis of India Data
Source: Nutrients. 2026 Jun 23;18(13):2045. doi: 10.3390/nu18132045 (PMC13363656; doi:10.3390/nu18132045)
Supplement: Supplementary file 1 [file nutrients-18-02045-s001.zip › nutrients-4337955-supplementary.pdf]

Table S1: Sample characteristics

	**Mean**	**Std. Err. [Linearized]**
Mean Child's dietary diversity score (CDD score)	3.26	.0133353
Mean HAZ score	-1.18	.0152984
**Gender of child**		
Male	51.48	.0023303
Female	48.52	.0039253
Age of child (months)	14.4	.0383442
Number of under-five children in household	1.69	.0039803
Mean Women's Dietary Diversity Score (WDD)	7.85	.0125484
Mean Women's highest year of education	4.24	.0081331
Mean time to water (minutes)	5.92	.0606742
Mean rainfall (mm)	1107.4	34.45007
Mean temperature [degree Celsius]	31.73	.0096763
**Water Availability Regions** [%]		
Low [LWAR]	18.38	.0017438
Medium [MWAR]	45.42	.0024342
High [HWAR]	36.18	.0021559
Safe disposal of child's stool (%)	42.61	.0010959
Access to improved toilet [%]	64.54	.002784
**Gender of household head**		
Male	85.03	.0019476
Female	14.97	.0020354
**Religion** [%]		
Hindu	81.79	.0029482
Muslim	13.75	.0028564
Christian	2.160	.0007312
Other	2.28	.0007667
**Asset class** [%]		
Lowest	.1967322	.0021529
Lower	.1930198	.0020147
Middle	.1953712	.0020508
Higher	.2077268	.0022796
Highest	.20715	.0024948
**Residence** [%]		
Rural	.7340676	.0025158
Urban	.2659324	.0025158
**Caste** [%]		
Schedule caste	.2465458	.0028731
Schedule tribe	.1063436	.0017958
OBC	.4575198	.0030711
General	.1895907	.0026411
